# Regulation of membrane ruffling by polarized STIM1 and ORAI1 in cortactin-rich domains

**DOI:** 10.1038/s41598-017-00331-4

**Published:** 2017-03-24

**Authors:** Aida M. Lopez-Guerrero, Patricia Tomas-Martin, Carlos Pascual-Caro, Thomas Macartney, Alejandro Rojas-Fernandez, Graeme Ball, Dario R. Alessi, Eulalia Pozo-Guisado, Francisco Javier Martin-Romero

**Affiliations:** 1 Department of Biochemistry and Molecular Biology, School of Life Sciences, University of Extremadura, Badajoz, 06006 Spain; 2 MRC- Protein Phosphorylation and Ubiquitylation Unit, School of Life Sciences, University of Dundee, Dundee, DD1 5EH Scotland UK; 3 Centre for Gene Regulation and Expression, School of Life Sciences, University of Dundee, Dundee, DD1 5EH Scotland UK; 4 Dundee Imaging Facility, School of Life Sciences, University of Dundee, Dundee, DD1 5EH Scotland UK; 5 Center for Interdisciplinary Studies on the Nervous System (CISNe) and Institute of Medicine, Universidad Austral de Chile, Valdivia, Chile; 6 Department of Cell Biology, School of Medicine, University of Extremadura, Badajoz, 06006 Spain

## Abstract

Cell motility and migration requires the reorganization of the cortical cytoskeleton at the leading edge of cells and extracellular Ca^2+^ entry is essential for this reorganization. However the molecular nature of the regulators of this pathway is unknown. This work contributes to understanding the role of STIM1 and ORAI1 in the promotion of membrane ruffling by showing that phospho-STIM1 localizes at the leading edge of cells, and that both phospho-STIM1 and ORAI1 co-localize with cortactin (CTTN), a regulator of the cytoskeleton at membrane ruffling areas. STIM1-KO and ORAI1-KO cell lines were generated by CRISPR/Cas9 genome editing in U2OS cells. In both cases, KO cells presented a notable reduction of store-operated Ca^2+^ entry (SOCE) that was rescued by expression of STIM1-mCherry and ORAI1-mCherry. These results demonstrated that SOCE regulates membrane ruffling at the leading edge of cells. Moreover, endogenous ORAI1 and overexpressed ORAI1-GFP co-immunoprecipitated with endogenous CTTN. This latter result, in addition to the KO cells’ phenotype, the preservation of ORAI1-CTTN co-localization during ruffling, and the inhibition of membrane ruffling by the Ca^2+^-channel inhibitor SKF96365, further supports a functional link between SOCE and membrane ruffling.

## Introduction

Cell motility is a complex cellular event that involves reorganization of the cytoskeleton. This reorganization includes the transient polarization of the cell to facilitate plasma membrane ruffling, a dynamic rearrangement of the cortical actin cytoskeleton which is required for the development of cellular protrusions, including lamellipodia and filopodia. In addition to protrusions, there is a transient formation of focal adhesions to stabilize the leading edge of migrating cells, followed by cell translocation and disassembly of focal adhesions at the rear part of the cell^1^. The signalling controlling these events is still a topic of study, but it is known that extracellular Ca^2+^ influx is essential for cell migration and for the positive-feedback cycle that maintains the leading-edge structure and ruffling activity^2, 3^. However, the mechanism that mediates this Ca^2+^ entry is still unknown. In this regard, store-operated Ca^2+^ entry (SOCE) has been pointed to as being involved in cell migration and metastasis^4, 5^. SOCE is an extracellular Ca^2+^ influx pathway controlled by STIM1 (stromal interaction molecule 1), an intraluminal Ca^2+^ sensor of the endoplasmic reticulum (ER) that activates store-operated calcium channels (SOCs) upon partial depletion of Ca^2+^ concentration within the ER^6–8^. Thus, numerous studies have reported that STIM1 function is required for cell migration^4, 9–14^. Indeed, both STIM1 and ORAI1, a plasma membrane SOC, are known to regulate focal adhesion turnover^4^. ORAI1 is a Ca^2+^ channel activated by STIM1 in the sense that ORAI1 mediates Ca^2+^ entry once STIM1 has become activated^15–17^. The mechanisms by which STIM1 activates ORAI1 are well known, and a detailed map of the protein-protein interaction sites is available and has been reviewed elsewhere^18, 19^. However, much less is known regarding the mechanism required for the STIM1 and ORAI1 localization in migrating cells.

STIM1 is transported throughout ER vesicles by a microtubule-dependent mechanism. EB1 (end-binding protein 1), a microtubule plus-end-tracking protein that regulates microtubule growth, binds directly to STIM1 through a sequence that includes residues 642–645 (Thr-Arg-Ile-Pro)^20^. To examine how STIM1 is transported to the front of migrating cells, Tsai *et al*. expressed YFP-STIM1 mutated at the EB1 binding sites (STIM1^I644N/P645N^). This mutant failed to polarize in migrating cells, although it is known to activate overall SOC influx to a similar degree as wild-type STIM1^13, 21^, indicating that the transport to the leading edge is microtubule dependent event. In this regard, in previous work, our group has found that STIM1 becomes phosphorylated at three ERK1/2 target sites, Ser575, Ser608, and Ser621, upon store depletion conditions triggered by thapsigargin, EGF, or IGF-1^21–23^. Once wild-type STIM1 is phosphorylated by ERK1/2, it dissociates from EB1, whilst a non-phosphorylatable mutant (STIM1^S575A/S608A/S621A^) remains bound to EB1 even after store depletion^21, 24^. Because dissociation from EB1 is required for the proper transport of STIM1 and subsequent binding to ORAI1, the Ser-to-Ala substitution mutant of STIM1 did not trigger full SOC influx in response to store depletion^21, 22^. These results highlighted the requirement of an EB1-dependent transport of STIM1, followed by the dissociation from microtubules at the target sites (ER-plasma membrane junctions) where STIM1 binds to SOC channels (such as ORAI1). However, the mechanism that regulates this last step needed for the polarized localization of STIM1 remains unclear.

Tsai *et al*. proposed that tyrosine kinase and phospholipase C signaling are restricted to the front of migrating endothelial leader cells, and that this polarization triggers local depletion of Ca^2+^ in the endoplasmic reticulum and local activation of STIM1 at the leading edge of migrating cells^13^. In that study, the authors argue that STIM1 acts indirectly on cell adhesion by enhancing local Ca^2+^ influx and ER Ca^2+^ refilling at the front of cells, supporting the hypothesis that higher SOCE at the front is required for the high turnover of focal adhesions. Nevertheless, there is a need for a model that explains the particular role of STIM1 and ORAI1 at the leading edge of migrating cells, in addition to the proposed role in recycling focal adhesions.

Because phosphorylation of STIM1 controls the activation of SOCE^21, 22^, as well as cell migration^9^, we hypothesized that STIM1 and ORAI1 might be directly involved in the regulation of Ca^2+^ entry at the leading edge of migrating cells. In particular, we wanted to study whether the proposed higher STIM1 activity at the leading edge might be revealed by higher levels of phospho-STIM1 in those areas. As a way to inquire into the cell migration role of STIM1-ORAI1, we also hypothesized that both pharmacological inhibition of Ca^2+^ entry and gene knock-out of STIM1 or ORAI1 would be sufficient to impair the reorganization of the cortical cytoskeleton required for the formation of lamellipodia and filopodia.

## Results

### Phospho-STIM1 is polarized in plasma membrane ruffling areas

**Figure 1:**
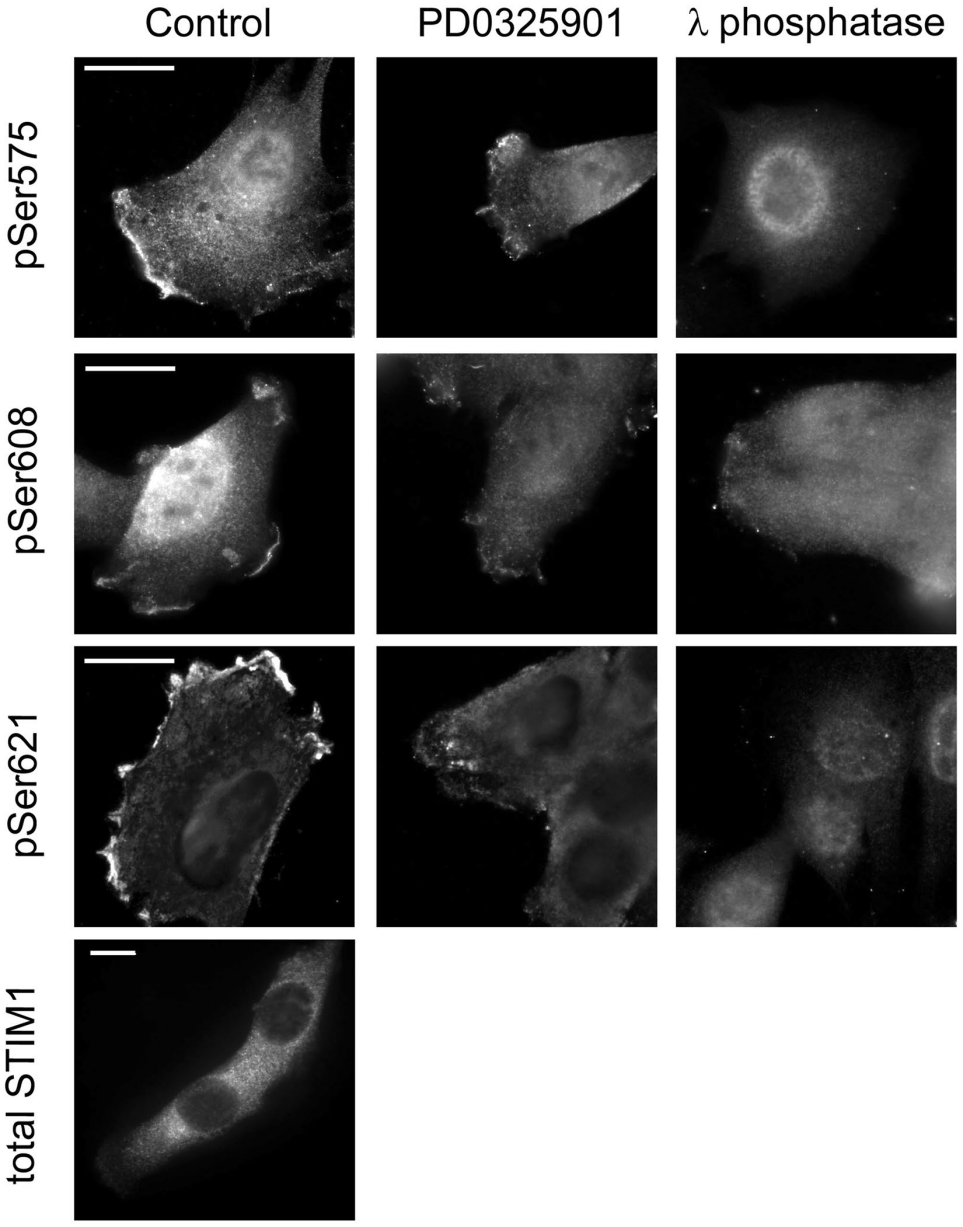
Immunolocalization of phospho-STIM1. C2C12 cells were plated onto collagen-coated glass coverslips, and fixed after 48 h in culture. Immunolocalization of phospho-STIM1 was performed for the three ERK1/2 target sites (pS575, pS605, and pS621) using phospho-specific antibodies and anti-sheep IgG labelled with Alexa Fluor 594 as a secondary antibody. Negative controls were performed by incubating cells with 0.5 μM PD0325901 for 20 min before fixation. Additional negative controls were performed, treating fixed cells with lambda phosphatase (10 U/μl) overnight at 30 °C. Total STIM1 was immunolocalized using an anti-STIM1 antibody and anti-rabbit IgG as a secondary antibody. Images are representative of at least 6 independent experiments. Bar = 10 μm.

Our group’s previous work using ectopic overexpression of Ser-to-Ala substitution mutants as well as phosphomimetic STIM1 mutants had shown that phosphorylation of STIM1 regulates cell migration in an adenocarcinoma cell model (Ishikawa cells)^9^. However, there is a major gap in knowledge regarding the localization of endogenous STIM1 phosphorylated at ERK1/2 target sites. To study this localization, in the present work we analysed phospho-STIM1 in C2C12 cells, a mouse mesenchymal cell line with high migration rates *in vitro*. We observed phospho-STIM1 (pSer575, pSer608, or pSer621) to mainly be found in the cell cortex, in areas of membrane ruffling (Fig. 1). Because the phosphorylation of STIM1 at Ser575, Ser608, and Ser621 is mainly mediated by ERK1/2 activity, inhibition of ERK1/2 with PD0325901 and direct treatment of fixed cells with lambda phosphatase were used as negative controls of this phospho-specific immunolocalization. The polarized localization was less evident for total endogenous STIM1 (Fig. 1), indicating that, in C2C12 cells, most of the phospho-STIM1 pool has a cortical distribution.

**Figure 2:**
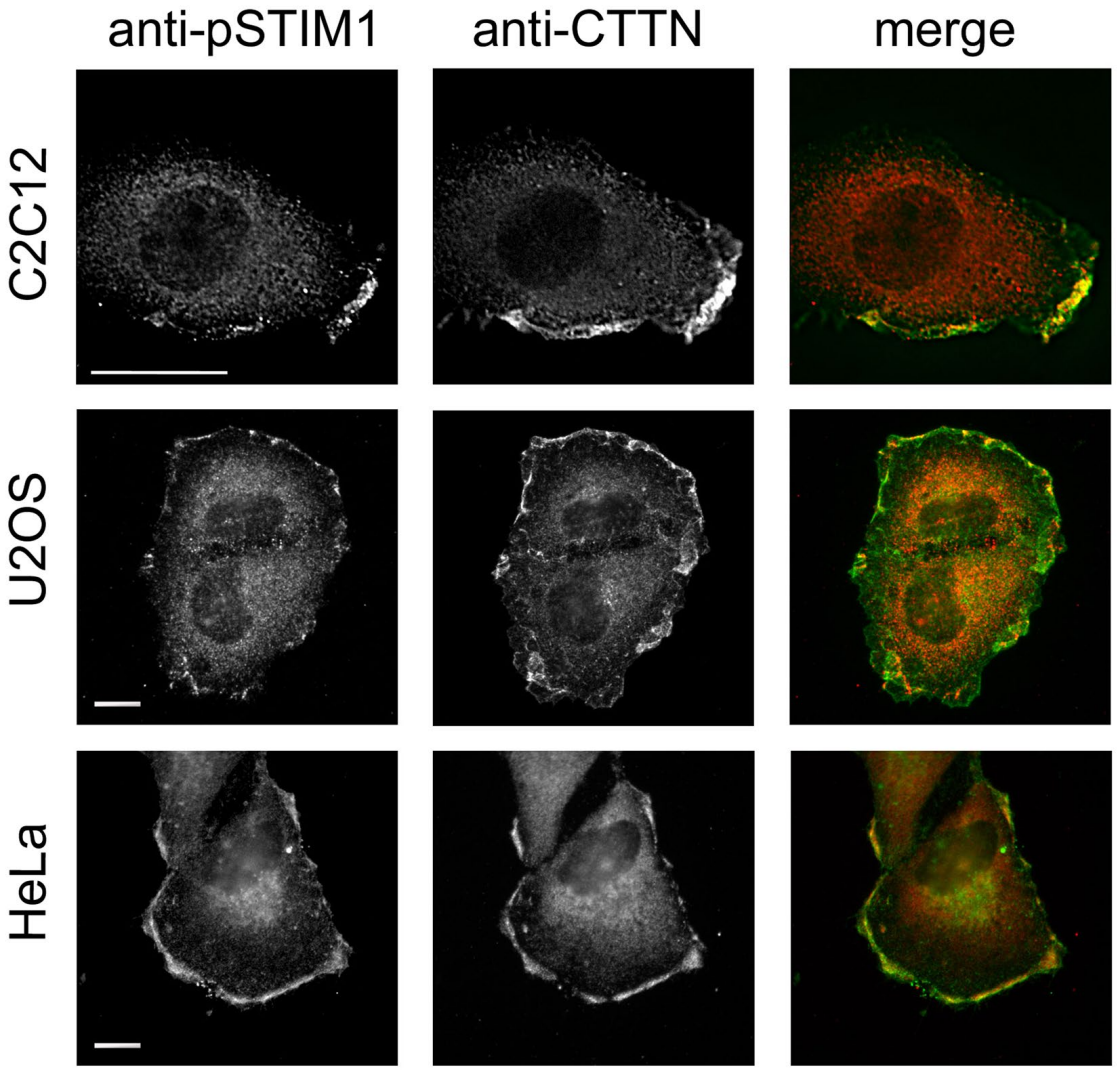
Immunolocalization of phospho-STIM1 and cortactin (CTTN). C2C12, HeLa, and U2OS cells were cultured for a minimum of 48 h as indicated in Materials and Methods. C2C12 were stimulated with FBS (20%), and HeLa and U2OS were stimulated with 50 ng/ml EGF for 10 min after 8–10 h of FBS-deprivation in RPMI medium. Fixed cells were used for the immunolocalization of phosho-STIM1 at Ser575 (pS575-STIM1) and cortactin (CTTN). Anti-sheep IgG labelled with Alexa Fluor 594 and anti-mouse IgG labelled with Alexa Fluor 488 were used as secondary antibodies for phospho-STIM1 and CTTN respectively. Images are representative of at least 10 independent experiments. Bar = 10 μm.

One of the best markers for the areas of membrane ruffling at the leading edge of migrating cells is the Src substrate cortactin (CTTN), a protein involved in the reorganization of the actin cytoskeleton in membrane ruffling areas^25–27^. CTTN is involved in cell shaping and in regulating cortical actin assembly and organization, and therefore plays a role in the formation of the membrane protrusions and lamellipodia which are required for cell migration. Interestingly, endogenous phospho-STIM1 has a high level of co-localization with endogenous cortactin, as we observed not just in C2C12 myoblasts, but also in the osteosarcoma cell line U2OS and in HeLa cells (Fig. 2). In this particular experiment, to promote cell migration, C2C12 cells were stimulated with FBS, and HeLa and U2OS cells were stimulated with epidermal growth factor, EGF (50 ng/ml). Because the phosphorylation of STIM1 at ERK1/2 target sites has been shown to be essential for the dissociation of STIM1 from EB1 and for the activation of SOC channels^9, 21–23, 28^, this polarized distribution of phospho-STIM1 suggests a role for Ca^2+^ influx through STIM1-dependent pathways in the remodelling of the cortical cytoskeleton required for lamellipodia and filopodia formation.

### STIM1 controls the dynamics of membrane ruffling

**Figure 3:**
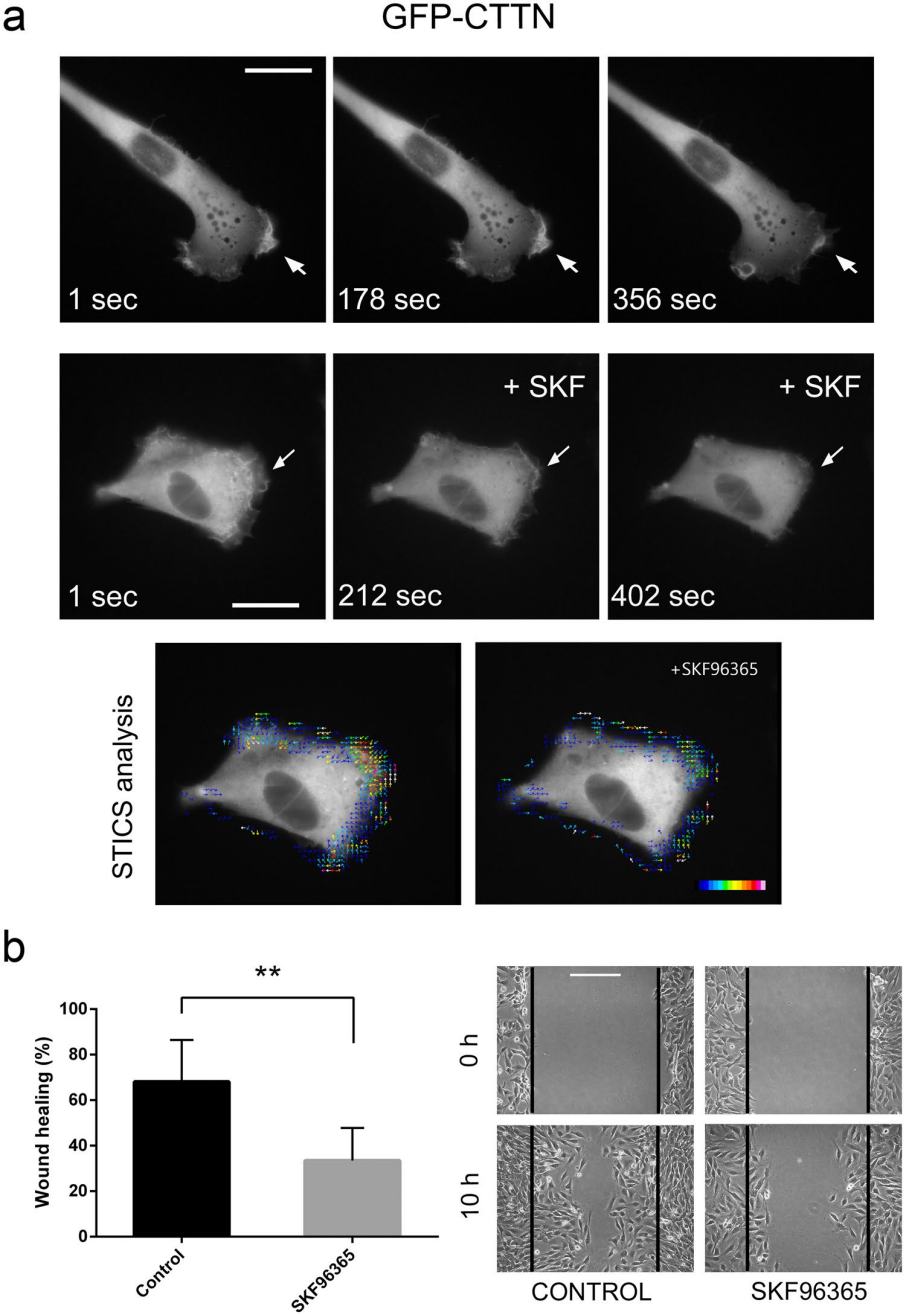
Dynamics of plasma membrane ruffling. (**a**) Top panels: C2C12 cells were transfected for the transient expression of GFP-CTTN, and GFP fluorescence emission was recorded under wide-field epifluorescence microscopy, with image acquisition every 2 sec for 5–7 min at 37 °C. During acquisition, cells were incubated in bicarbonate-free Leibovitz’s L-15 medium supplemented with 10% FBS. Areas of membrane ruffling are marked with arrows. Middle panels: Cells were transfected for the transient expression of GFP-CTTN and observed for GFP emission. At time = 200 sec, SKF96365 (10 μM) was added to cell culture medium (+SKF), and the observation extended for 3 min. Full time-lapse sequences are shown in Supplementary Movies 1 and 2. The images and time-lapses are representative of three independent experiments. Bar = 20 μm. Bottom panels: Spatiotemporal image correlation spectroscopy (STICS) analysis of GFP-CTTN fluorescence was performed before and after addition of SKF to assess direction and velocity of membrane ruffling, which is shown in a pseudocolor scale as overlaid vectors. (**b**) Cell migration was evaluated by a wound-healing assay. Cells were plated onto collagen-coated glass coverslips and after scratching the cell monolayer (time = 0) the culture was extended for 10 hours. SKF96365 (10 μM) was added to the culture medium at time = 0. Cells were photographed and quantitative image analysis was performed with ImageJ software. The data are presented as the mean ± s.d. of at least 28 independent images from 4 experiments. Bar = 200 μm. Statistical analysis was done using the unpaired t-test. **p < 0.01.

We therefore undertook the study of the influence of STIM1 on the dynamics of membrane ruffling as a way to inquire into the role of STIM1-dependent Ca^2+^ entry in cell migration. For this purpose, we transfected C2C12 cells with GFP-CTTN to monitor membrane ruffling. Supplementary Movie 1 is a time-lapse sequence of the GFP-CTTN dynamics in C2C12 cells (also represented in Fig. 3a), showing intense membrane ruffling. The addition of the SOC inhibitor SKF96365 (10 μM) to the culture medium, intended to block Ca^2+^ entry, led to rapid cessation of membrane ruffling (Fig. 3a, and Supplementary Movie 2), supporting a causal role for Ca^2+^ entry through a STIM1-dependent pathway in the reorganization of the cortical cytoskeleton at the leading edge of migrating cells. As a consequence of this reduced membrane ruffling in the presence of SKF96365, we observed a significant reduction of cell migration in C2C12 cells in wound-healing assays for cells treated with the SOC inhibitor (Fig. 3b).

**Figure 4:**
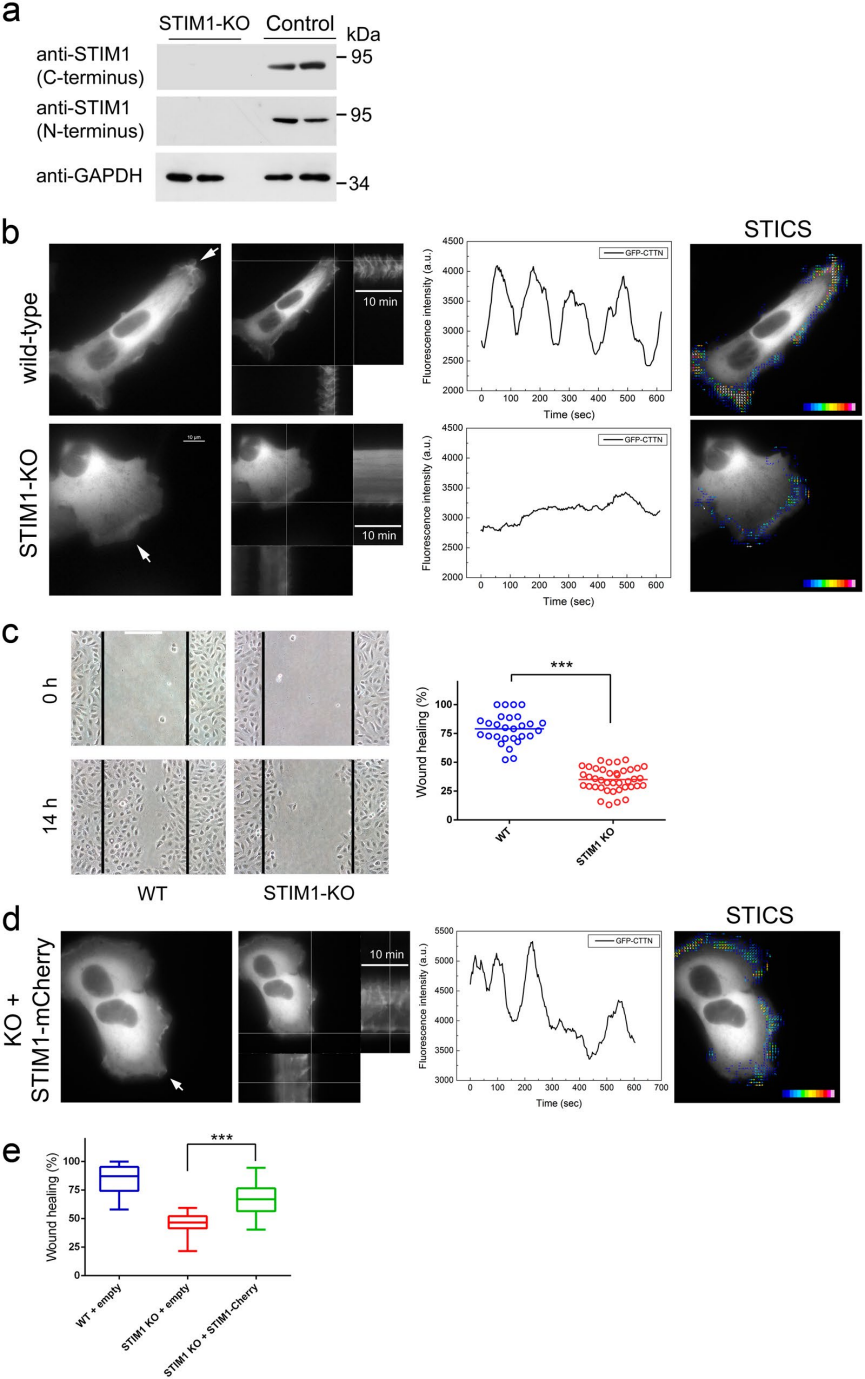
Knock-out of STIM1 expression by CRISPR/Cas9 D10A gene editing. Gene editing was performed in U2OS cells following the strategy presented in Supplementary Fig. 1. (**a**) STIM1 expression was assessed using antibodies against C-terminal and N-terminal epitopes. GAPDH was used as loading control. (**b**) Top panels. 1: wild-type cells were transfected for the transient expression of GFP-CTTN, and monitored for 10 min at 37 °C (image acquisition every 3 sec). Assay medium was Leibovitz’s L-15 + 10% FBS. 2: Orthogonal projections of the areas marked show the fluorescence intensity over the time (10 min) (kymograph). 3: GFP-CTTN fluorescence of the selected area in panel 2 was evaluated to assess membrane ruffling. 4: STICS analysis is shown as overlaid vectors on a pseudocolor scale. The figure is representative of 32 recordings from 4 independent assays. Full time-lapse is given in Supplementary Movie 3. Bottom panels. 1: STIM1-KO cells were transfected for the transient expression of GFP-CTTN, and monitored as indicated above (Panels 2–4). The figure is representative of 22 independent assays. Full time-lapse is given in Supplementary Movie 4. (**c**) Cell migration was evaluated by a wound-healing assay. Cell monolayer was scratched (time = 0), and the culture was extended for 14 hours in DMEM + 10% FBS. Quantitative image analysis was performed with ImageJ. Bar = 200 μm. Scatter plot depicts the mean of at least 28 independent images from 4 experiments. (**d**) STIM1-KO cells were transfected for transient expression of GFP-CTTN and STIM1-mCherry, and mCherry-positive cells were selected to monitor GFP fluorescence (CTTN) as in panel b. Assay medium was Leibovitz’s L-15 + 10% FBS. The figure is representative of 21 recordings from 3 independent assays. Full time-lapse is shown in the Supplementary Movie 5. (**e**) Cell migration of STIM1-KO cells transfected for the expression of STIM1-mCherry was assessed by a wound-healing assay. Control cells (wild-type or KO) were transfected for the expression of empty vector. The box-plot depicts data from 3 independent experiments (n = 70, wild-type; n = 60, STIM1-KO; n = 100, STIM1-KO + STIM1-mCherry). Statistical analysis was done using the unpaired *t*-test. ***p < 0.001.

Because these results revealed that STIM1 was directly involved in the dynamics of plasma membrane ruffling, we genetically modified U2OS cells to target STIM1 expression by gene knock-out (KO) using the CRISPR/Cas9 genome editing system. U2OS cells were transfected with specific vectors harbouring Cas9 D10A (Cas9 nickase, or Cas9n), reducing the risk for unspecific double-strain breaks as performed elsewhere^29^, and with specific guide RNAs (gRNAs) designed to target the 3 known transcriptional variants of human STIM1 (which encode for proteins NP_001264890.1, NP_001264891.1, and NP_003147.2) (Supplementary Fig. 1a). For the selected positive clone of cells, the modification consisted of a 31 bp + 17 bp deletion. This generated a translational frameshift and premature stop codons, leading to the knock-out of *STIM1* gene expression, as revealed by an immunoblot using two different antibodies (Fig. 4a). Accordingly, these STIM1-KO cells showed a striking decrease in the level of SOCE triggered by thapsigargin or EGF (Supplementary Fig. 1b,c), an ideal experimental condition with which to demonstrate the role of STIM1 and SOCE in plasma membrane ruffling and cell migration. We then studied whether overexpression of ectopic STIM1 would rescue the loss of function, so as to be able to reject any off-target effects during the generation of the KO cell lines. For that purpose we transfected STIM1-KO cells for the transient expression of STIM1-mCherry, and observed full recovering of SOCE in transfected cells only, whereas Ca^2+^ entry was minimal in untransfected cells (Supplementary Fig. 1b).

U2OS wild-type (wt) and STIM1-KO cells were transfected for the transient expression of GFP-CTTN, and monitored under wide-field epifluorescence microscopy to assess plasma membrane ruffling dynamics. An evaluation of the fluorescence of GFP-CTTN over 10 min of assay demonstrated an intense membrane ruffling in wild-type cells (Fig. 4b and Supplementary Movie 3), similarly to what we observed in C2C12 cells (Fig. 3). Interestingly, membrane ruffling was greatly diminished in STIM1-KO cells (Fig. 4b and Supplementary Movie 4). The ruffling is shown in Fig. 4 as a kymograph at the orthogonal projections in both panels, for wild type and KO cells. We also show the spatiotemporal image correlation spectroscopy (STICS) analysis to evaluate direction and velocity of this ruffling, as performed elsewhere^30^. As a result of this reduced membrane ruffling, STIM1-KO U2OS cells showed a significantly reduced cell migration in a wound-healing assay compared to control cells (Fig. 4c), confirming the key role of STIM1 in the generation of membrane protrusions, an essential event for cell migration.

The loss of membrane ruffling in STIM1-KO cells was rescued by ectopic overexpression of STIM1-mCherry (Fig. 4d and Supplementary Movie 5). Moreover, ectopic overexpression of STIM1 normalized cell migration levels, assessed by wound-healing assays (Fig. 4e). Taken together, these results suggest that the loss of membrane ruffling in KO cells was not due to off-target effects, and provide further support for a direct role of STIM1 in the regulation of membrane ruffling.

### ORAI1 is polarized in cortactin-rich areas and controls the dynamics of membrane ruffling

**Figure 5:**
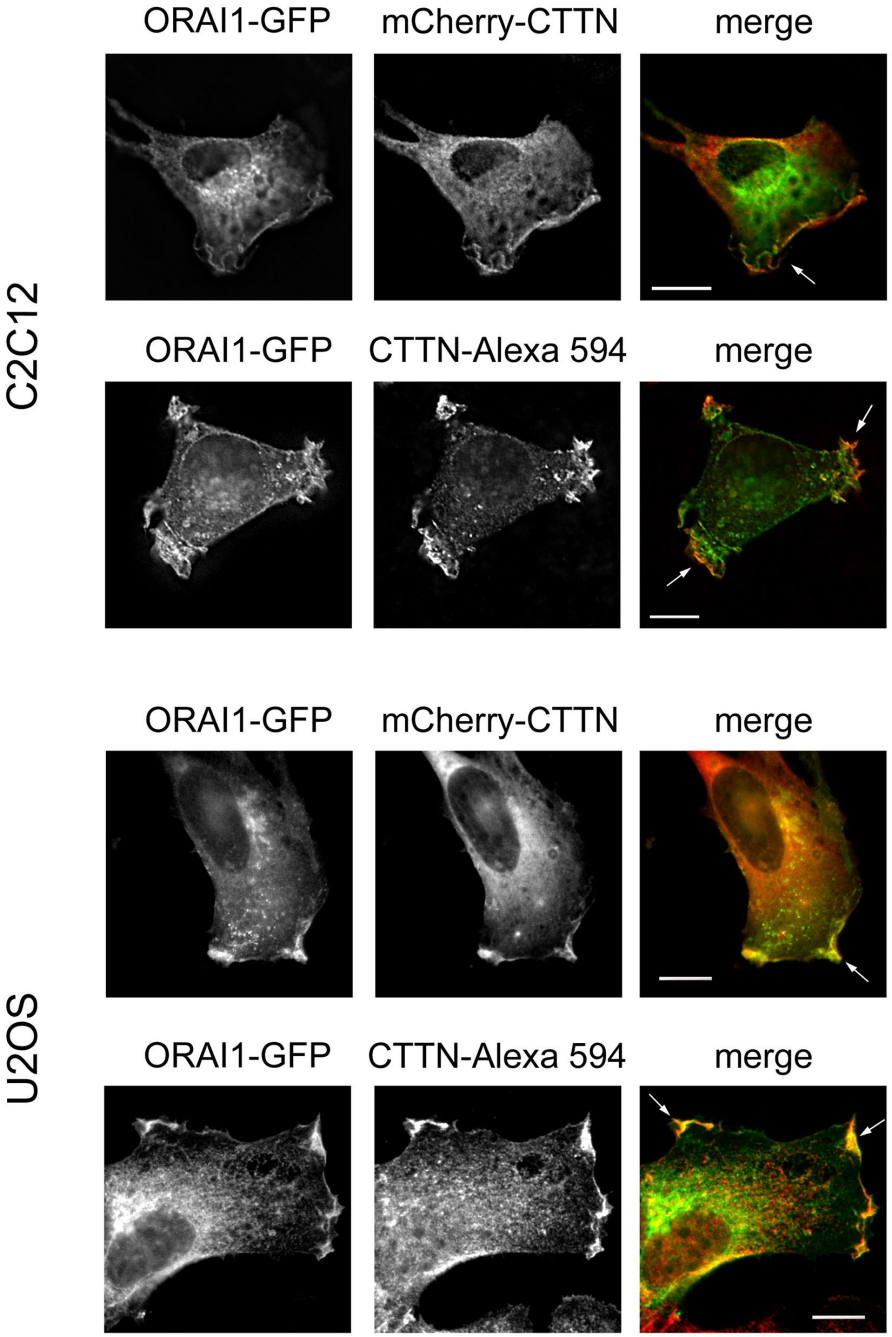
Co-localization of ORAI1 and CTTN. Top panels: C2C12 and U2OS were transfected for the transient transfection of ORAI1-GFP and mCherry-CTTN, and visualized under wide field epifluorescence microscopy. As in Fig. 2, C2C12 cells were stimulated with FBS (20%), and U2OS cells were stimulated with 50 ng/ml EGF for 10 min after 8–10 h of FBS-deprivation in RPMI medium. Images are representative of at least 6 independent experiments for every cell line. Bar = 10 μm. Bottom panels: ORAI1-GFP transfected cells were assessed for endogenous CTTN localization with a mouse monoclonal anti-cortactin antibody and anti-mouse IgG labelled with Alexa Fluor 594. Images are representative of at least 5 independent experiments for both cell lines. Bar = 10 μm.

Because phospho-STIM1 was found to be a marker of cortactin-rich areas, and therefore a marker for the leading edge of migrating cells, and due to the fact that the most important effector of STIM1 is the plasma membrane Ca^2+^ channel ORAI1, we hypothesized that the leading edge of migrating cells would be ORAI1 enriched. Consequently, we transfected cells with ORAI1-mCherry to monitor its localization using endogenous CTTN or GFP-CTTN as markers of the leading edge (Fig. 5). We found that ORAI1 and endogenous CTTN show a high level of co-localization in C2C12 and U2OS cells, which is further evidence for a high SOCE activity at these CTTN-rich sites. Similar results were found for ORAI1 and overexpressed GFP-CTTN, validating the use of labelled CTTN.

**Figure 6:**
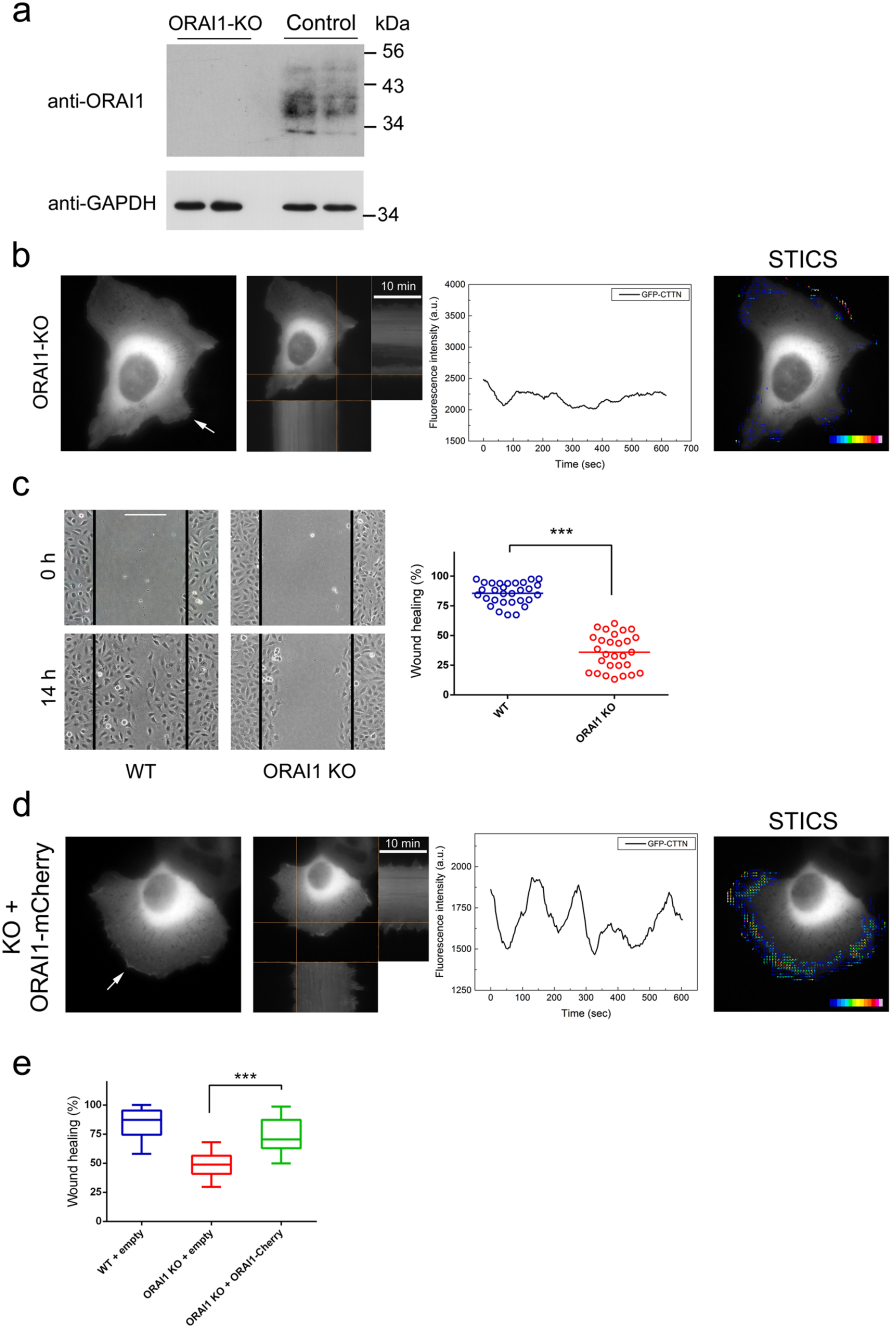
Knock-out of ORAI1 expression by CRISPR/Cas9 D10A gene editing. Gene editing was performed in the U2OS cell line following the strategy shown in Supplementary Fig. 2. (**a**) The selected clone of cells was assessed for ORA1 expression by immunoblot. GAPDH was used as loading control. (**b**) Panel 1: ORAI1-KO cells were transfected for the transient expression of GFP-CTTN, and monitored for 10 min at 37 °C (image acquisition every 3 sec). Assay medium was Leibovitz’s L-15 medium +10% FBS. Panel 2: Orthogonal projections show the kymograph of the fluorescence intensity over the time of assay (10 min). Panel 3: GFP-CTTN fluorescence of the selected area is shown to indicate the frequency and extent of membrane ruffling. Panel 4: STICS analysis is shown as overlaid vectors on a pseudocolor scale. Full time-lapse sequence is given in Supplementary Movie 6. The figure is representative of 20 recording from 3 independent assays. (**c**) Cell migration was evaluated in U2OS ORAI1-KO cells by a wound-healing assay. Cells were plated onto collagen-coated glass coverslips, the cell monolayer was scratched (time = 0), and the culture extended for 14 hours in DMEM + 10% FBS. Quantitative image analysis was performed with ImageJ software. Bar = 200 μm. Data are presented as the mean and the scatter distribution of 28 results from 4 independent experiments. (**d**) ORAI1-KO cells were transfected for transient expression of GFP-CTTN and ORAI1-mCherry, and mCherry-positive cells were selected to monitor GFP fluorescence (CTTN) as indicated in panel b. The assay medium was Leibovitz’s L-15 + 10% FBS. The figure is representative of 19 recordings from 3 independent assays. The full time-lapse sequence is given in Supplementary Movie 7. (**e**) Cell migration of ORAI1-KO cells transfected for the expression of ORAI1-mCherry was assessed by a wound-healing assay, as in panel c. Control cells (wild-type and KO cells) were transfected for the expression of empty vector. The box-plot depicts data from 3 independent experiments (n = 70 for wild-type; n = 59 for ORAI1-KO; n = 100 for ORAI1-KO + ORAI1-mCherry). Statistical analysis was done using the unpaired t-test. ***p < 0.001.

The results so far pointed to ORAI1 as a possible major regulator of membrane ruffling given: (i) the observation that SKF96365 inhibits the ruffling (Fig. 3); (ii) the particular localization of phospho-STIM1 and ORAI1 (Figs 1, 2 and 5); and (iii) STIM1-KO cells did show an impaired ruffling and cell migration (Fig. 4). We therefore decided to examine the role of this channel in the cortical reorganization of the cytoskeleton by generating ORAI1-KO cells using the CRISPR/Cas9 system and specific gRNAs designed to target the 2 known transcriptional variants of human ORAI1 (protein NP_116179.2 and a shorter predicted variant with Uniprot accession number A0A087WTK9). Following CRISPR targeting and genotyping, the final selected KO clone was found to contain 16 bp + 14 bp deletions in the *ORAI1* locus leading to translation frameshift and premature stop codons in both alleles (Supplementary Fig. 2a). The loss of ORAI1 protein expression was confirmed by immunoblot (Fig. 6a). ORAI1-KO cells showed a markedly low level of SOCE triggered by thapsigargin or EGF (Supplementary Fig. 2b,c), indicating that ORAI1 is the most predominant member of the ORAI family in controlling SOCE in U2OS cells. As we had done with STIM1-KO cells, we studied whether the overexpression of ectopic ORAI1 rescued the wild-type phenotype so as to be able to reject off-target effects. Transfecting ORAI1-KO cells with ORAI1-mCherry, we observed levels of SOCE similar to those found in wild-type cells, confirming the specificity of the strategy to knock-out *ORAI1* gene (Supplementary Fig. 2).

ORAI1-KO U2OS cells were transfected for the expression of GFP-CTTN to monitor plasma membrane ruffling and cytoskeleton reorganization at the lamellipodia formation sites. The results demonstrate a striking loss of ruffling activity and protrusions formation in ORAI1-KO cells (Fig. 6b and Supplementary Movie 6), compared to control cells (see Fig. 4b, top panels). In addition, in wound-healing assays we observed a significant reduction in cell migration compared to control cells (Fig. 6c), further confirming the role of the axis STIM1-ORAI1 as the major Ca^2+^ entry pathway for the reorganization of the cortical cytoskeleton and the generation of protrusions and lamellipodia. As we had done with the STIM1-KO cells, we transfected ORAI1-KO cells with ORAI1-mCherry, and observed full recovery of membrane ruffling in only those transfected cells (Fig. 6d and Supplementary Movie 7). Also, cell migration in wound-healing assays was normalized by overexpression of ORAI1 in KO cells (Fig. 6e), confirming the hypothesis that STIM1 and ORAI1 promote membrane ruffling and regulate cell migration.

**Figure 7:**
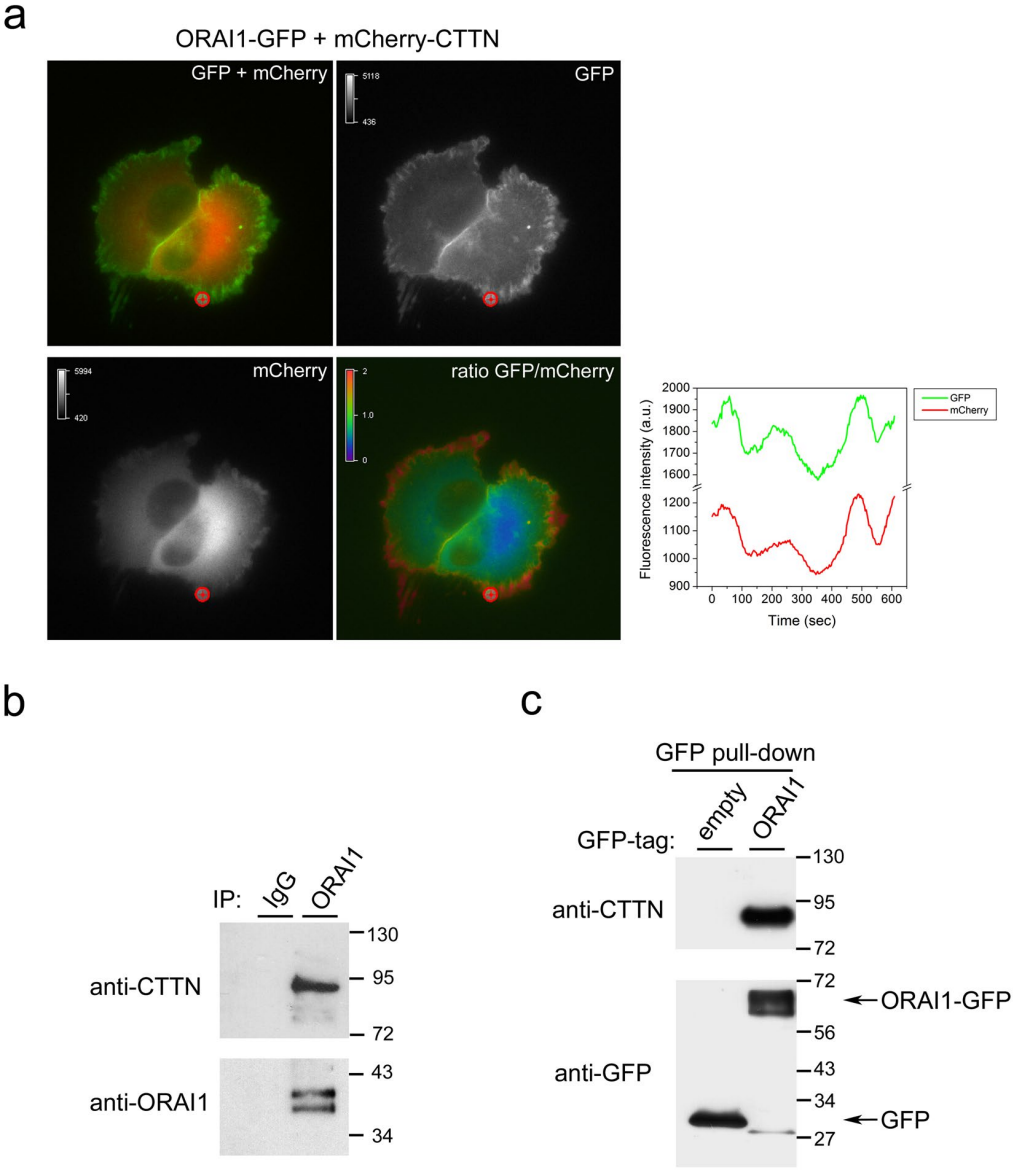
ORAI1-CTTN co-localization and co-precipitation. (**a**) U2OS wild-type cells were transfected for the transient expression of ORAI1-GFP/mCherry-CTTN and monitored for both tags in Leibovitz’s L-15 medium supplemented with 10% FBS. Emission of fluorescence was recorded every 3 sec for 10 min, and images were split in both channels (GFP and mCherry). The GFP/mCherry ratio was calculated from single channels, and is shown on a pseudocolor scale (ratio values 0–2). A selected area was assessed for the variation of fluorescence intensity, shown in the line graph. The full time-lapse sequence is given in Supplementary Movie 8. (**b**) Endogenous ORAI1 was immunoprecipitated with an anti-ORAI1 antibody conjugated to agarose beads (lane 2). The level of immunoprecipitated ORAI1 was assessed with an anti-ORAI1 antibody (bottom panel), and the level of co-immunoprecipitated CTTN was evaluated with an anti-CTTN antibody (top panel). As a negative control, lysates from the same experimental conditions were incubated with preimmune serum and agarose beads (lane 1). Blots are representative of 3 independent experiments with 3 different cultures. (**c**) U2OS cells transiently expressing GFP (lane 1) or ORAI1-GFP (lane 2) were lysed to pull-down GFP-tagged ORAI1 with GFP-Trap beads. The level of endogenous CTTN bound to ORAI1-GFP was evaluated by immunoblot using an anti-CTTN antibody (top panel), and the level of pulled-down GFP or ORAI1-GFP was evaluated with an anti-GFP antibody (bottom panel). Blots are representative of 4 independent experiments with 4 different cultures.

Because the ruffling areas are highly dynamic, we were interested in monitoring ORAI1-CTTN dynamics at these sites with ORAI1-GFP/mCherry-CTTN double labelling and monitoring live cells for both tags to study whether the two proteins show mutually dependent movement in this area. Supplementary Movie 8 shows a 10 min time-lapse sequence with the dynamics of both fluorescent proteins in U2OS cells. Figure 7a shows a frame from the time-lapse experiment with split channels (GFP plus mCherry channels and single channels) and the calculated ratio GFP/mCherry on a pseudocolor scale. Analysis of the GFP/mCherry fluorescence indicates that spiking of fluorescence is identical for both tags in the ruffling area, and therefore the ratio is preserved, suggesting a possible functional or even physical link between CTTN and ORAI1. To study further this possible interaction we immunoprecipitated endogenous ORAI1 from U2OS cells and observed specific co-immunoprecipitation of endogenous CTTN (Fig. 7b), a result that was confirmed with cells overexpressing ORAI1-GFP. When we pulled-down GFP from these cells, we observed specific co-precipitation of endogenous CTTN only when ORAI1-GFP was precipitated, but no co-precipitation for GFP-(empty vector)-expressing cells (Fig. 7c).

## Discussion

The role of Ca^2+^ signaling on cell migration is a point of intense debate since the description of STIM1 and ORAI1 as two of the most important regulators of Ca^2+^ entry in eukaryotic cells. A significant advance in this field was the report by Yang *et al*. that the knockdown of STIM1 or ORAI1 by RNA interference slows cell migration *in vitro* and inhibits the metastasis of MDA-MB-231 cells in nude mice^4^. These results, together with the inhibition of focal adhesion turnover and tumour progression in mice by SKF96365, a SOC inhibitor, suggested that SOCE underlies a significant part of the Ca^2+^ entry required for cell migration. It is unclear, however, what might be the intracellular Ca^2+^-sensitive mechanism that regulates focal adhesion assembly or disassembly. Some aspects of this regulation have been proposed. An example is the participation of the Ca^2+^-dependent protease calpain^31^, which could increase disassembly rates by cleaving talin at focal adhesion sites, a proteolysis that could lead to disassembly of other focal adhesion components, such as paxillin, vinculin, and zyxin^32^. In this regard, it is also known that EGF stimulates the phosphorylation of proline-rich tyrosine kinase 2 beta (PTK2B or PYK2) at Tyr402, and of the focal adhesion kinase (FAK) at Tyr397. These two events are required for focal adhesion turnover, and both are inhibited by the knockdown of STIM1 expression^10^. However, disassembly of focal adhesion plaques is not part of the initial steps in the timeline of cell migration. On the contrary, it seems to be a late action for Ca^2+^, and Ca^2+^-dependent disassembly of focal adhesions should be important at the rear part of the migrating cell, where there are higher rates of cytoskeleton retraction. It is therefore possible that the diffusion of cytosolic Ca^2+^, or the front-to-rear axis gradient of intracellular Ca^2+^ could be regulating differential events in a Ca^2+^ concentration specific manner. In this regard, the aforementioned reports do not contain information regarding the localization of STIM1-ORAI1, information that is critical to being able to define the spatio-temporal regulation of Ca^2+^ signaling.

Thus, the study of the localization of STIM1 and ORAI1 in migrating cells might explain how Ca^2+^ signaling diffuses or acts locally in restricted sites. The strong polarization of the Ca^2+^ signaling mediators in migrating cells is demonstrated in the present results by the finding that there is phospho-STIM1 enrichment at the leading edge of cells, where cortactin (CTTN) acts a marker of the reorganization of the cortical cytoskeleton known as membrane ruffling. Together with Arp2/3, cortactin, is a regulator of the branching of F-actin, and they both play key roles in the formation of protrusions (filopodia and lamellipodia), and consequently are found at the front of migrating cells^25–27^. Given that phospho-STIM1 has been described as the active form of STIM1 in activating Ca^2+^ entry^21, 22^, the present results support a model in which there is high activity of STIM1 and STIM1-dependent Ca^2+^ entry at the leading edge. The finding that endogenous phospho-STIM1 is a marker for the leading edge of migrating cells clearly defines a new concept which might be useful in the study of cell motility and migration. It also provides further evidence for greater STIM1 activity at the leading edge, in agreement with the results reported by Tsai *et al*. when they used ectopically expressed YFP-STIM1 and a CFP-tagged ER marker^13^.

Phosphorylation of STIM1 at ERK1/2 sites is attained during stimulation of cells with activators of the MAPK (mitogen-activated protein kinases) pathway such as EGF^9^ or IGF-1^23^, and we have reported that this phosphorylation facilitates the dissociation of STIM1 from microtubules (i.e., EB1). Because of the receptor tyrosine kinase (RTK) enrichment at the front of migrating cells, a model is plausible in which activation of RTKs by ligands could lead to partial depletion of ER Ca^2+^ levels because of the activation of the phosphoinositide pathway^9, 23^. This partial and local emptying of the ER, together with the ERK1/2-dependent phosphorylation of STIM1, would increase local and transient Ca^2+^ pulses. This model requires the participation of a Ca^2+^ channel activated by STIM1, and the present results are the first to show ORAI1-GFP enrichment at the leading edge of cells, co-localizing and co-precipitating with CTTN, and supporting the proposed mechanism identifying the Ca^2+^ pathway that regulates cell migration. Again, our proposal fits well with the reported greater PMCA (plasma membrane Ca^2+^-ATPase) activity at the front^13^, because a high rate of cytosolic Ca^2+^ spikes has to be expected at these sites, and therefore a high rate of Ca^2+^ extrusion to counteract those transitory increases of [Ca^2+^]_i_. However, it is still unclear how STIM1 and ORAI1 remain sequestered at these sites.

Our results give a full explanation for earlier observations suggesting that an extracellular Ca^2+^ influx is required for a positive feedback at the leading edge of spontaneously polarized macrophages^3^. Although the molecular pathway for this Ca^2+^ influx may vary from one cell type to another, our findings demonstrate that STIM1-ORAI1 mediate this Ca^2+^ influx in membrane ruffling. In addition to the findings regarding the physical location of phospho-STIM1 and ORAI1 that circumscribes SOCE at a restricted site, the knock-out of *STIM1* and *ORAI1* genes for all the known transcripts reveals that both proteins are fully required for membrane ruffling, and that the impaired ruffling can be rescued by ectopic expression of STIM1 or ORAI1. We have monitored membrane ruffling by recording GFP-CTTN fluorescence dynamics. These dynamics are the result of the continuous protrusion of actin cytoskeleton in the form of filopodia and F-actin branching to increase the actin network density (lamellipodia). The inhibition of Ca^2+^ entry by SKF96365 demonstrated that these dynamics require Ca^2+^ entry, but this was not as specific as the knockout of particular genes, and, with CRISPR/Cas9 genome editing, we created KO cells with a minimal impact on genomic stability (indels of 31 bp or smaller). To the best of our knowledge, this is the first reported strategy to KO *STIM1* and *ORAI1* loci using this method.

Finally, the high value of the ratio between ORAI1-GFP and mCherry-CTTN fluorescence in live cells indicates that the two proteins are strongly co-localized in migrating cells during the formation of protrusions, and that there is no significant loss of this co-localization in the subsequent retractions. Moreover, we monitored specific co-immunoprecipitation between endogenous ORAI1 and endogenous CTTN, as well as strong and specific co-immunoprecipitation between overexpressed ORAI1-GFP and CTTN. This result strongly suggests that ORAI1 and CTTN are part of the same macromolecular complex. A study of the mechanisms and regulation of this interaction lay beyond the objectives of the present work, but our results do open the possibility of studying the molecular mechanisms that regulate cell migration with the description of STIM1 and ORAI1 as new partners in this process. They also suggest that these two proteins act in cooperation with other regulators of the cytoskeleton such as CTTN, and possibly with other regulators of cell motility such as the small GTPase Rac1 due to its well-known role as activator of CTTN^33, 34^, something that needs further study.

## Materials and Methods

### Chemicals

Flp-In T-REx U2OS cells were kindly provided by Dr. Gopal Sapkota (University of Dundee); HeLa and C2C12 myoblasts were from ECACC (European Collection of Cell Cultures) and distributed by Sigma-Aldrich (St. Louis, MO, USA); All cell lines were tested for contamination before carrying out the experiments shown in this report. PD0325901 was purchased from Axon Medchem (Groningen, The Netherlands); Fura-2-acetoxymethyl ester (fura-2-AM) was from Calbiochem, a brand belonging to Merck Millipore (Darmstadt, Germany); Thapsigargin (Tg) and SKF96365 were from Abcam Biochemicals (Cambridge, UK); Polyethylenimine was purchased from Polysciences, Inc (Eppelheim, Germany); Collagen, type I, was purchased from Sigma-Aldrich (St. Louis, MO, USA). GFP-Trap resin was from Chromotek GmbH (Planegg-Martinsried, Germany).

### Antibodies

Rabbit polyclonal anti-STIM1 antibody was from ProSci Inc. (Poway, CA, USA). Mouse monoclonal anti-STIM1 antibody was from BD Biosciences (San Jose, CA, USA). Antibodies against phospho-Ser575-STIM1, phospho-Ser608-STIM1, and phospho-Ser621-STIM1 were produced in collaboration with the Division of Signal Transduction Therapy (DSTT), University of Dundee (Dundee, UK), as described elsewhere^9, 21, 23^. Mouse monoclonal anti-ORAI1 (clone G-2) antibody, and anti-ORAI1 (clone G-2) antibody coupled to agarose beads were from Santa Cruz Biotechnology (Heidelberg, Germany). Mouse monoclonal anti-cortactin (clone 4F11) was purchased from Merck Millipore (Darmstadt, Germany). Mouse monoclonal anti-GAPDH antibody was from AbCam (Cambridge, UK). Rabbit anti-GFP antibody was from Cell Signaling Technology (Danvers, MA, USA). All secondary HRP- and Alexa Fluor-labelled antibodies were from ThermoFisher Scientific (Waltham, MA USA).

### DNA constructs

For transient transfection, Stim1 cDNA (mouse Stim1, NM_009287) was cloned into the pmCherry-N1 vector (Life Technologies) as an *Xho*I-*Bam*HI vector by adding those restriction sites to the Stim1 cDNA by PCR. The construct for the transient transfection of GFP-tagged mouse cortactin (GFP-CTTN) was provided by Dr. Anna Huttenlocher^35^ and distributed by Addgene (plasmid #26722). Plasmid mCherryC1-Cortactin was provided by Christien Merrifield (Addgene plasmid #27676). Orai1 cDNA (Human Orai1, NM_032790.3) was cloned into a pcDNA5FRT/To-GFP or into a pcDNA5FRT/To-mCherry vector as a *Bam*HI-*Not*I insert. The construct for the transient transfection of ORAI1-mCherry (construct DU52310) can be requested on our reagents website (https://mrcppureagents.dundee.ac.uk/).

Site-directed mutagenesis was performed with the QuikChange Multi Site-Directed Mutagenesis Kit (Agilent Technologies, Santa Clara, CA, USA). DNA constructs used for transfection were purified from *E. coli* DH5α using Qiagen Plasmid kits according to the manufacturer's protocol. All DNA constructs were verified by DNA sequencing at the DNA Sequencing Service of the University of Dundee (www.dnaseq.co.uk), or at the Sequencing Facility of STAB, University of Extremadura. Transfection of cells with DNA constructs was performed with 1–5 μg plasmid DNA per 10-cm dish and polyethylenimine in serum-containing medium, 24 h prior to the beginning of the experiments.

### Culture of cells

C2C12 myoblasts were cultured in Dulbecco's modified Eagle's medium (DMEM) with 20% (v/v) foetal bovine serum (FBS), 2 mM L-glutamine, 100 U/ml penicillin, and 0.1 mg/ml streptomycin in a humidified atmosphere of air/CO_2_ at 37 °C. HeLa and U2OS cells were cultured in DMEM with 10% (v/v) FBS, 2 mM L-glutamine, 100 U/ml penicillin, and 0.1 mg/ml streptomycin, in a humidified atmosphere of air/CO_2_ at 37 °C. Cells were studied under stimulation with FBS or after FBS deprivation for 8–10 h in RPMI medium followed by stimulation with 50 ng/ml EGF. In all cases cell culture dishes and glass coverslips were treated with collagen type I solution (0.01%) for a minimum of 30 min at 37 °C.

### Generation of genetically modified cells using CRISPR/Cas9 genome editing

Analysis of the *STIM1* locus (ENSG00000167323) showed three verified transcriptional variants; NM_001277961.1 (known as STIM1L), NM_001277962.1 (for STIM1S), and NM_003156.3 (for canonical STIM1). While the first coding exon shared between the published transcripts was exon 3, there remained some evidence of a shorter 512 residue variant initiating further downstream. Exon 5 was therefore chosen as the CRISPR target site. The guide pair (sense 5′-(G)AGATGACAGACCGGAGTCAT and antisense 5′-(G)AGTCCCTGTCATGGTGGTGT) was subsequently identified using the Sanger Institute CRISPR webtool (http://www.sanger.ac.uk/htgt/wge/find_crisprs). Complementary oligos with *Bbs*I compatible overhangs were designed according to the Zhang method^36^ and these dsDNA guide inserts ligated into *Bbs*I-digested target vectors; the antisense guide was cloned into the spCas9 D10A expressing vector pX335 (Addgene Plasmid #42335) and the sense guide into the puromycin selectable plasmid pBABED P U6 (University of Dundee)^37, 38^. Cells were co-transfected with 1 μg of each plasmid using polyethylenimine in a 10-cm dish. Following 24 h of recovery and a further 48 h of puromycin selection (2 μg/ml), the transfection was repeated, and cells subjected to a further round of puromycin selection to enrich for transfectants. The cell pool was subsequently single-cell sorted by FACS, and clones were analysed for STIM1 depletion by immunoblotting and sequencing. Genomic DNA was isolated, and the region surrounding the target site of the guide RNAs amplified by PCR (forward primer: 5′-CAAGAGCTAGAAGTGTTCCTGGG; reverse primer: 5′-CTTTGGTTTCCATGGCACAGC). The resulting PCR products were subcloned using the StrataClone Blunt PCR Cloning Kit (Agilent Technologies) and ten colonies picked for each clonal line and sequenced to verify indels. PCR products are mixed following CRISPR due to differences between the targeted alleles, but it has been found that, in practice, analysis of 10 clones from a given clonal line is sufficient to verify the allele population^39^. Sequencing of the exon 5 PCR fragments from the STIM1-KO cells revealed a 31 + 17 base-pair deletion confirming the successful KO of the *STIM1* locus.

Analysis of the ORAI1 locus (ENSG00000276045) showed two transcriptional variants: NM_032790.3 and a shorter predicted variant A0A087WTK9 (Uniprot accession number). An optimal guide pair (sense 5′-(G)CCAAGCTTAAAGCCTCCAGC and antisense 5′-(G)GCTCAAGTAGAGCTTGCGCC) was identified and chosen to target the first common coding exon shared between these published transcripts. dsDNA guide inserts were then cloned into the nickase system, transfected and CRISPR-targeted cells subsequently enriched for via puromycin selection as described above for the STIM1 KO. Genotyping was performed using primers flanking the CRISPR target site (F: 5′-TCACCTACCCGGACTGGATCG and R: 5′-AGCAGCCGGGACAGTAGAGG) with PCR products being shotgun cloned (StrataClone Blunt PCR Cloning Kit) and sequenced. Ten colonies were again picked to obtain data for the two alleles, and sequencing of the chosen ORAI1-KO cell line revealed a mix of 16 + 14 base-pair insertions confirming the complete KO of the *ORAI1* locus.

DNA constructs for the generation of STIM1-KO (constructs DU52282, DU52301) and ORAI1-KO cells (constructs DU52285, DU52304) can be requested on our reagents website (https://mrcppureagents.dundee.ac.uk/).

### Immunoprecipitation and immunoblot

Immunoblot for STIM1 was performed as indicated previously^9, 21–23^. Cells were lysed in the following buffer: 50 mM Tris-HCl (pH 7.5), 1 mM EGTA, 1 mM EDTA, 1% (w/v) Nonidet P40, 1 mM sodium orthovanadate, 50 mM sodium fluoride, 5 mM sodium pyrophosphate, 0.27 M sucrose, 0.1% (v/v) 2-mercaptoethanol, 1 mM benzamidine, and 0.1 mM phenylmethylsulfonyl fluoride. After lysis, samples were clarified with 0.75–1 ml of ice-cold lysis buffer/dish and centrifugation at 20,000 g for 15 minutes at 4 °C. Then samples were sonicated with five 10-second pulses using a Branson Digital Sonifier with a setting of 45% amplitude. Protein concentration was determined using the Coomassie Protein Assay Reagent.

Lysates (10–40 μg) were subjected to electrophoresis on polyacrylamide gels (4–12% acrylamide) and subsequent electroblotting to nitrocellulose membranes. Membranes were blocked for 1 h at room temperature (RT) in blocking buffer: TBS-T (Tris-buffered saline buffer, pH 7.5, with 0.2% Tween-20) containing 10% (w/v) non-fat milk. Then the membranes were incubated overnight with 1 μg/ml anti-STIM1 antibody (ProSci, Inc.) or 1 μg/ml anti-STIM1 antibody (BD Biosciences) diluted in blocking solution at 4 °C, washed, and then incubated with anti-rabbit or anti-mouse IgG horseradish peroxidase (HRP)-conjugated secondary antibody (1:20 000 dilution) for 1 hour at RT in blocking solution.

Immunoblot for ORAI1 was performed by incubating membranes overnight at 4 °C with 1 μg/ml anti-ORAI1 antibody diluted in blocking buffer, followed by incubation with anti-mouse HRP-conjugated secondary antibody (1:10 000 dilution). Immunoblots for GFP and CTTN were performed by incubating membranes with 1.23 μg/ml anti-GFP antibody, or 1 μg/ml anti-CTTN antibody respectively, in both cases diluted in blocking buffer, and followed by incubation with anti-rabbit or anti-mouse HRP-conjugated secondary antibodies (1:20 000 dilution). In all cases luminol substrate was added, and membranes were exposed for 1–3 min to chemiluminescence films. The signal of developed films was quantified by volumetric integration using Image J.

ORAI1-GFP was purified with GFP-Trap agarose beads. Beads (5 μl) were added to clarified cell lysates (2.5 mg), followed by incubation for 1 h at 4 °C with gentle shaking. GFP-Trap beads were washed with 1 ml lysis buffer containing 0.15 M NaCl and twice with 50 mM Tris–HCl, 0.1 mM EGTA, pH 7.5. Proteins were eluted from the beads by the addition of 7 μl NuPAGE-LDS sample buffer to the beads. Eluted proteins were reduced by the addition of 10 mM DTT followed by heating at 72 °C for 10 min. To immunoprecipitate untagged endogenous ORAI1, 0.5 mg of clarified cell lysate was incubated with 5 μl anti-ORAI1 antibody covalently conjugated to agarose beads, for 1 h at 4 °C with gentle shaking. The immunoprecipitate was washed with 1 ml lysis buffer containing 0.15 M NaCl and twice with 1 ml 50 mM Tris–HCl, 0.1 mM EGTA, pH 7.5. Protein elution was performed as indicated above and samples were subjected to electrophoresis on 4–12% polyacrylamide gels.

### Immunolocalization and wide-field epifluorescence microscopy

Immunolocalization of endogenous cortactin was performed on cells fixed with 4% paraformaldehyde in PBS for 15 min at RT. Permeabilization was performed with 0.2% Triton X-100 and blocking with 3% fish skin gelatin in PBS + 0.2% Tween-20 for 30 min at room temperature. Cells were incubated 1 hour with the mouse monoclonal anti-cortactin antibody at 1:100 dilution. Anti-mouse IgG labeled with Alexa Fluor 594 (or Alexa Fluor 488) was used as a secondary antibody (diluted 1:1000, 20 min at RT).

Fixation and permeabilization of cells for the immunolocalization of phospho-STIM1 was performed as indicated above. Phospho-STIM1 antibodies were pre-blocked with their specific non-phosphorylated peptides, and then cells were incubated at 4 °C for 12–16 h with the anti-phospho-Ser575-STIM1, anti-phospho-Ser608-STIM1, or anti-phospho-Ser621-STIM1 antibodies (10 μg/ml in all cases)^9, 21, 23, 24^. Cells were then incubated with sheep IgG antibody labelled with Alexa Fluor 594, diluted 1:1000 in blocking solution at RT for 20 min. Coverslips were mounted onto glass slides with Hydromount (National Diagnostics). Images of fixed cells were taken on a Nikon Ti-E inverted epifluorescence microscope with a Plan Apochromat 100× (NA 1.45) oil immersion objective.

GFP- and mCherry-tagged proteins were live cell imaged at 37 °C in an UNO-Okolab stage incubator. Bicarbonate-free Leibovitz’s L-15 medium supplemented with 10% FBS was used during recordings. The excitation settings were a 480/30 nm excitation filter, 535/40 nm emission filter and 505 nm dichroic mirror for GFP, and a 562/40 nm excitation filter, together with a 641/75 emission filter, and 593 nm dichroic mirror for mCherry (Semrock). Acquisition time was typically 20–40 msec per frame, with time intervals of 3 sec, for 5–10 min. Focus was continuously adjusted with the perfect focusing system (PFS) of the Nikon Ti-E microscope.

### Cytosolic free calcium concentration measurement

Cytosolic free calcium concentration ([Ca^2+^]_i_) was measured basically as described elsewhere^22, 40, 41^, in fura-2-AM-loaded cells, using an inverted microscope Nikon Ti-E equipped with micro-incubation platform DH-40i (Warner Instruments). Excitation fluorescence wavelengths were selected with 340/26 and 387/11 nm filters (Semrock), and emission fluorescence with a 510/10 nm filter. All measurements were performed at 35–36 °C. Excitation/emission conditions were controlled by the NIS-Elements AR software. Depletion of Ca^2+^ stores was triggered by incubating cells with 1 μM Tg in Ca^2+^-free HBSS with the following composition: 138 mM NaCl; 5.3 mM KCl; 0.34 mM Na_2_HPO_4_; 0.44 mM KH_2_PO_4_; 4.17 mM NaHCO_3_; 4 mM Mg^2+^ (pH 7.4). SOCE was measured by monitoring the increase of the [Ca^2+^]_i_ after the addition of 2 mM CaCl_2_ to the Tg-containing medium.

### Image data analysis

Kymographs are the average of fluorescence intensity across a 3-pixel width of the selected scan line. Spatiotemporal image correlation spectroscopy (STICS) analysis^42^ was used to assess direction and velocity of GFP-labelled cortactin in the periphery of cells by using the ImageJ plugin STICS map v2 (Stowers Institute for Medical Research in Kansas City, MO, USA). Background correction in fluorescence images was performed using ImageJ or NIS-Elements AR software.

### Statistical analysis of data

Statistical analyses were done using the unpaired *t*-test. Differences between groups of data were taken statistically significant for *p* < 0.05. The *p*-values are represented as follows: *p < 0.05, **p < 0.01, and ***p < 0.001.

## Electronic supplementary material

Suppl Files 1_2 and legends to supplementary figures

Supplementary Movie 1

Supplementary Movie 2

Supplementary Movie 3

Supplementary Movie 4

Supplementary Movie 5

Supplementary Movie 6

Supplementary Movie 7

Supplementary Movie 8
