# Supplementary material for: Regulation of membrane ruffling by polarized STIM1 and ORAI1 in cortactin-rich domains
Source: Sci Rep. 2017 Mar 24;7:383. doi: 10.1038/s41598-017-00331-4 (PMC5428229; doi:10.1038/s41598-017-00331-4)
Supplement: Supplementary file 1 — Suppl Files 1_2 and legends to supplementary figures [file 41598_2017_331_MOESM1_ESM.pdf]

SUPPLEMENTARY INFORMATION

Title: REGULATION OF MEMBRANE RUFFLING BY POLARIZED STIM1 AND ORAI1 IN CORTACTIN-RICH DOMAINS

Authors: Aida M. López-Guerrero, Patricia Tomas-Martin, Carlos Pascual-Caro, Thomas Macartney, Alejandro Rojas-Fernandez, Graeme Ball, Dario R. Alessi, Eulalia Pozo-Guisado, Francisco Javier Martin-Romero.

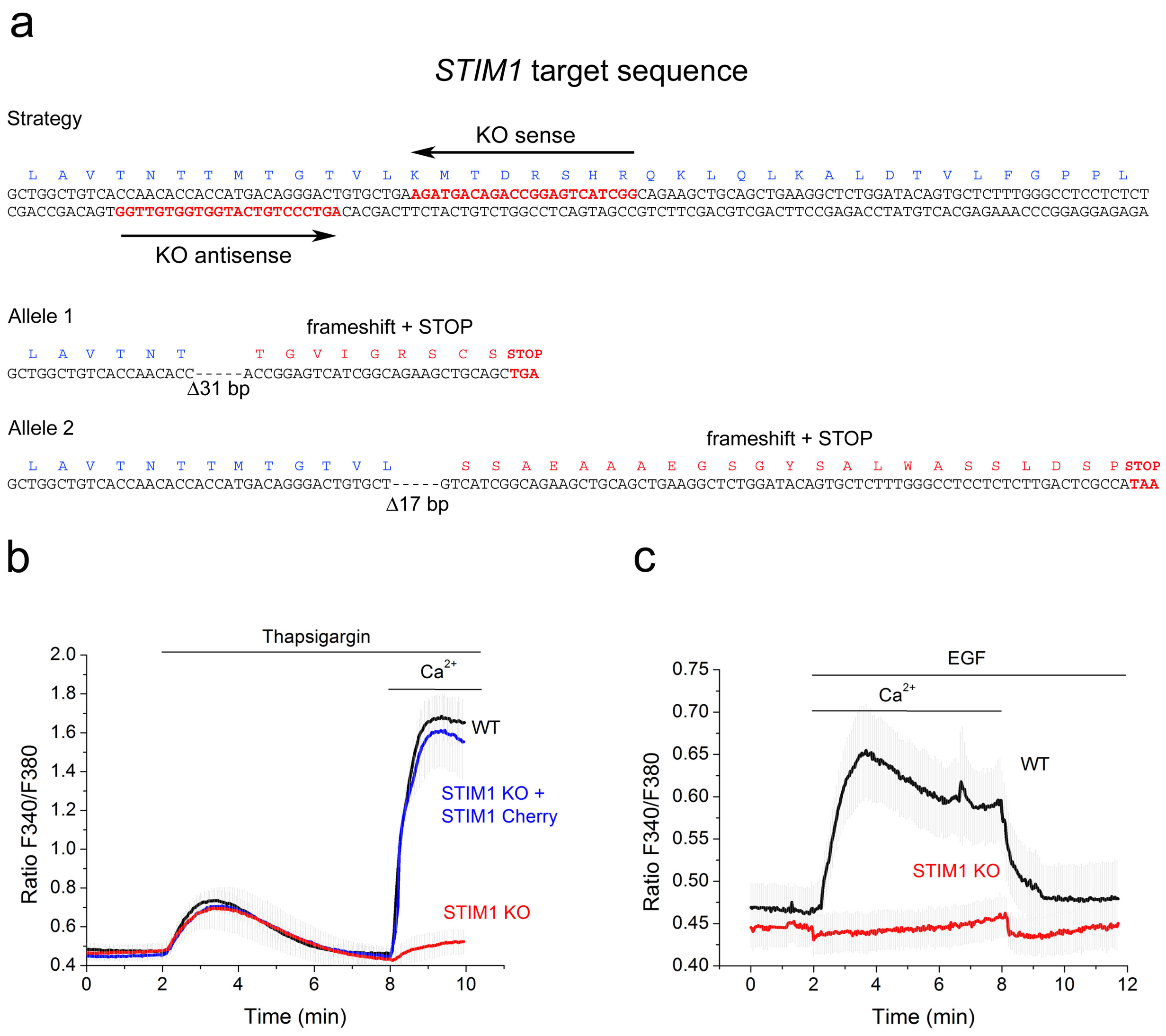

a
STIM1 target sequence
Strategy
KO sense
L A V T N T T M T G T V L K M T D R S H R Q K L Q L K A L D T V L F G P P L
GCTGGCTGTCACCAACACCACCATGACAGGGACTGTGCTGAAGATGACAGACCGGAGTCATCGGCAGAAGCTGCAGCTGAAGGCTCTGGATACAGTGCTCTTTGGGCCTCCTCTCT
CGACCGACAGTGGTTGTGGTGGTACTGTCCCTGACACGACTTCTACTGTCTGGCCTCAGTAGCCGTCTTCGACGTCGACTTCCGAGACCTATGTCACGAGAAACCCGGAGGAGAGA
KO antisense
Allele 1
frameshift + STOP
L A V T N T T G V I G R S C S STOP
GCTGGCTGTCACCAACACC-----ACCGGAGTCATCGGCAGAAGCTGCAGCTGA
Δ31 bp
Allele 2
frameshift + STOP
L A V T N T T M T G T V L S S A E A A A E G S G Y S A L W A S S L D S P STOP
GCTGGCTGTCACCAACACCACCATGACAGGGACTGTGCT-----GTCATCGGCAGAAGCTGCAGCTGAAGGCTCTGGATACAGTGCTCTTTGGGCCTCCTCTCTTGACTCGCCATAA
Δ17 bp
b
Thapsigargin
Ca2+
WT
STIM1 KO +
STIM1 Cherry
STIM1 KO
Ratio F340/F380
Time (min)
2.0
1.8
1.6
1.4
1.2
1.0
0.8
0.6
0.4
0
2
4
6
8
10
c
EGF
Ca2+
WT
STIM1 KO
Ratio F340/F380
Time (min)
0.75
0.70
0.65
0.60
0.55
0.50
0.45
0.40
0
2
4
6
8
10
12

Suppl Fig 1

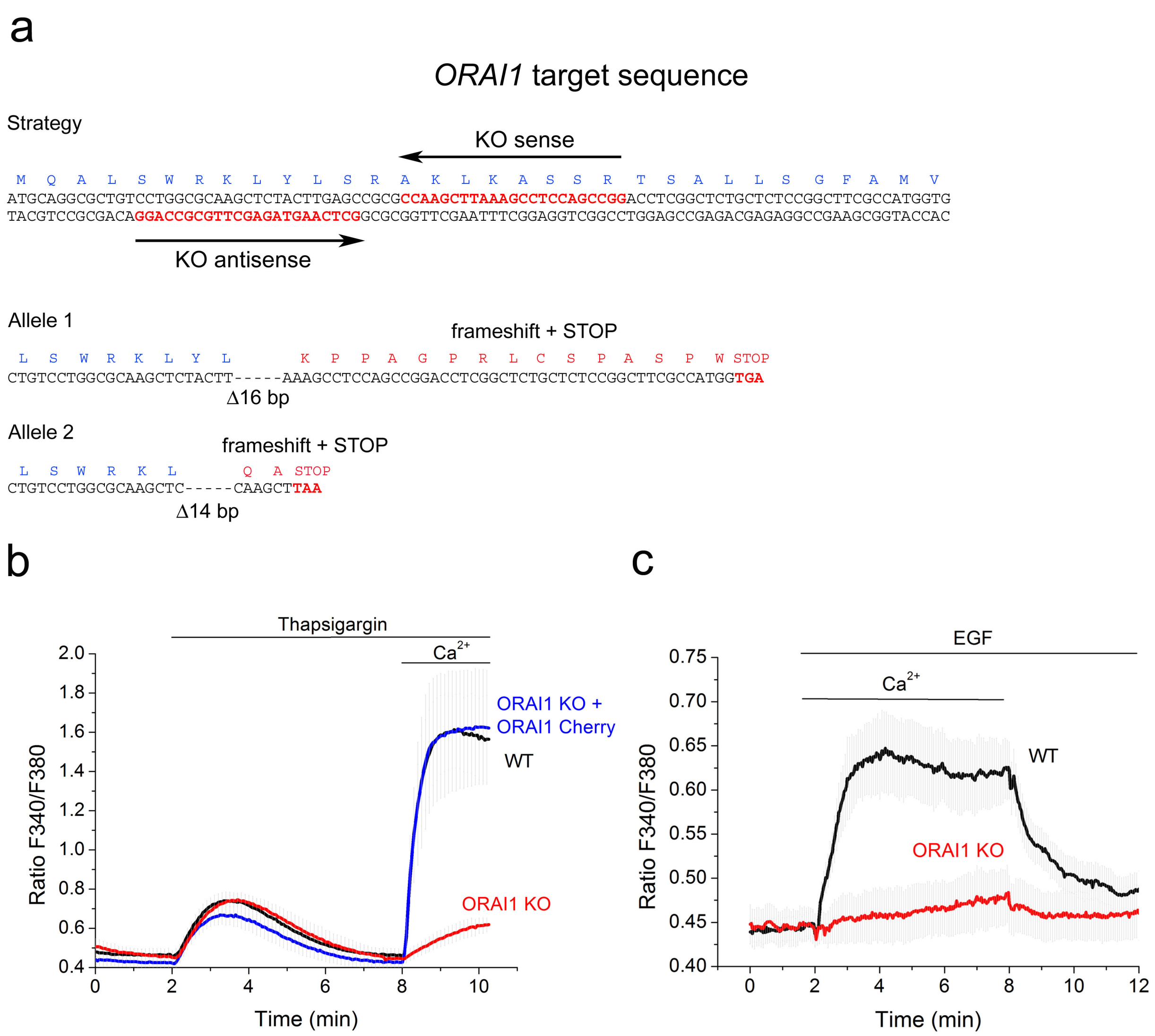
a
ORAI1 target sequence
Strategy
KO sense
M Q A L S W R K L Y L S R A K L K A S S R T S A L L S G F A M V
ATGCAGGCGCTGTCCTGGCGCAAGCTCTACTTGAGCCGCGCCAAGCTTAAAGCCTCCAGCCGGACCTCGGCTCTGCTCTCCGGCTTCGCCATGGTG
TACGTCCGCGACAGGACCGCGTTCGAGATGAACTCGGCGCGGTTCGAATTTCGGAGGTCGGCCTGGAGCCGAGACGAGAGGCCGAAGCGGTACCAC
KO antisense
Allele 1
frameshift + STOP
L S W R K L Y L K P P A G P R L C S P A S P W STOP
CTGTCCTGGCGCAAGCTCTACTT-----AAAGCCTCCAGCCGGACCTCGGCTCTGCTCTCCGGCTTCGCCATGGTGA
Δ16 bp
Allele 2
frameshift + STOP
L S W R K L Q A STOP
CTGTCCTGGCGCAAGCTC-----CAAGCTTAA
Δ14 bp
b
Thapsigargin
Ca2+
ORAI1 KO +
ORAI1 Cherry
WT
ORAI1 KO
Ratio F340/F380
2.0
1.8
1.6
1.4
1.2
1.0
0.8
0.6
0.4
0
2
4
6
8
10
Time (min)
c
EGF
Ca2+
WT
ORAI1 KO
Ratio F340/F380
0.75
0.70
0.65
0.60
0.55
0.50
0.45
0.40
0
2
4
6
8
10
12
Time (min)

Suppl Fig 2

LEGENDS TO SUPPLEMENTARY FILES

Supplementary Figure 1. Knock-out of STIM1 expression by CRISPR/Cas9 D10A gene editing. (a) Strategy for the knock-out of *STIM1* locus in U2OS cells. The pair of guide RNAs designed to trigger a double nick at exon 5 of *STIM1* locus is in red font. The indels found in U2OS were a 31 bp + 17 base-pair deletion. The resulting translational frameshifting and premature stop codons are shown in red font. (b) Cells were assessed for a Ca^{2+} entry assay using thapsigargin to trigger the emptying of intracellular Ca^{2+} stores. Fura-2-loaded cells were incubated in Ca^{2+}-free HBSS (assay medium) at 35ºC, and 1 μM thapsigargin (Tg) was added to the cells for 6 min. Ca^{2+} (2 mM $CaCl_2$) was added to the cells to evaluate the extent of Ca^{2+}-entry in KO cells (red line) and control cells (black line). In parallel, STIM1-KO cells were transfected for the transient expression of STIM1-mCherry to evaluate the rescue of the wild-type phenotype (blue line). Data are presented as the mean ± s.d. of 3 independent experiments (n > 25 cells). (c) Fura-2-loaded cells were incubated in Ca^{2+}-free HBSS at 35ºC (baseline), and 50 ng/ml EGF + 2 mM $CaCl_2$ was added to the cells to evaluate the extent of Ca^{2+}-entry in STIM1 KO cells (red line) and control cells (black line). After 6 min, assay medium was replaced by Ca^{2+}-free HBSS + 50 ng/ml EGF to monitor the drop in Ca^{2+} entry in the absence of extracellular Ca^{2+}. Data are presented as the mean ± s.d. of 3 independent experiments (n = 35 cells for KO and n= 40 cells for wild-type).

Supplementary Figure 2. Knock-out of ORAI1 expression by CRISPR/Cas9 D10A gene editing. (a) Strategy for the knock-out of *ORAI1* locus in U2OS cells. The pair of guide RNAs designed to trigger a double nick at exon 1 of *ORAI1* locus is in red font. The indels found in U2OS were 16 + 14 base-pair deletions. The resulting translational frameshifting and premature stop codons are in red font. (b) Cells were assessed for a Ca^{2+} entry assay using thapsigargin to trigger the emptying of intracellular Ca^{2+} stores. Fura-2-loaded cells were incubated in Ca^{2+}-free HBSS (assay medium) at 35ºC, and 1 μM thapsigargin (Tg) was

added to the cells for 6 min. Ca^{2+} (2 mM $CaCl_2$) was added to the cells to evaluate the extent of Ca^{2+}-entry in KO cells (red line) and control cells (black line). In parallel, ORAI1-KO cells were transfected for the transient expression of ORAI1-mCherry to evaluate the rescue of the wild-type phenotype (blue line). Data are presented as the mean ± s.d. of 3 independent experiments (n > 30 cells). (c) Fura-2-loaded cells were incubated in Ca^{2+}-free HBSS at 35ºC (baseline), and 50 ng/ml EGF + 2 mM $CaCl_2$ was added to the cells to evaluate the extent of Ca^{2+}-entry in ORAI1 KO cells (red line) and control cells (black line). After 6 min, assay medium was replaced by Ca^{2+}-free HBSS + 50 ng/ml EGF to monitor the drop in Ca^{2+} entry in the absence of extracellular Ca^{2+}. Data are presented as the mean ± s.d. of 3 independent experiments (n = 38 cells for KO and n= 42 cells for wild-type).

Supplementary Movie 1. Dynamics of plasma membrane ruffling in C2C12 cells. The time-lapse sequence depicts the recorded GFP-CTTN fluorescence in C2C12 cells. GFP fluorescence emission was recorded for 6 min at 37ºC, with image acquisition every 2 sec. Other details are given in Figure 3.

Supplementary Movie 2. Dynamics of plasma membrane ruffling in cells treated with SKF96365. The time-lapse sequence depicts the recorded GFP-CTTN fluorescence in C2C12 cells before and after addition of 10 μM SKF96365 to show the slowing of membrane ruffling in the presence of this SOC inhibitor. Other details are given in Figure 3.

Supplementary Movie 3. Dynamics of plasma membrane ruffling in wild-type U2OS cells. U2OS cells were transfected for the transient expression of GFP-CTTN, and monitored under epifluorescence microscopy for 10 min, with image acquisition every 3 sec. Other experimental conditions are given in the legend to Figure 4.

Supplementary Movie 4. Dynamics of plasma membrane ruffling in STIM1-KO U2OS cells. STIM1-KO U2OS cells were transfected for the transient expression of GFP-CTTN,

and monitored under epifluorescence microscopy for 10 min, with image acquisition every 3 sec. Other experimental conditions are given in the legend to Figure 4.

Supplementary Movie 5. Rescue of phenotype in STIM1-KO cells transfected with STIM1. STIM1-KO U2OS cells were transfected for the transient expression of STIM1-mCherry and GFP-CTTN. mCherry positive cells were monitored for GFP fluorescence emission for 10 min, with image acquisition every 3 sec. Other experimental conditions are given in the legend to Figure 4.

Supplementary Movie 6. Dynamics of plasma membrane ruffling in ORAI1-KO U2OS cells. ORAI1-KO cells were transfected for the transient expression of GFP-CTTN, and monitored under epifluorescence microscopy for 10 min, with image acquisition every 3 sec. Other experimental conditions are given in the legend to Figure 6.

Supplementary Movie 7. Rescue of phenotype in ORAI1-KO cells transfected with ORAI1. ORAI1-KO U2OS cells were transfected for the transient expression of ORAI1-mCherry and GFP-CTTN. mCherry positive cells were monitored for GFP fluorescence emission for 10 min, with image acquisition every 3 sec. Other experimental conditions are given in the legend to Figure 6.

Supplementary Movie 8. Dynamics of ORAI1-CTTN localization in U2OS cells. U2OS cells were transfected for the transient expression of mCherry-CTTN and ORAI1-GFP, and monitored under epifluorescence microscopy for 10 min, with image acquisition every 3 sec. Other experimental conditions are given in the legend to Figure 7.
